# Adverse childhood experiences and internalizing symptoms: the moderating role of neural responses to threat

**DOI:** 10.1016/j.ynstr.2025.100740

**Published:** 2025-06-10

**Authors:** Carola Dell’Acqua, Claudio Imperatori, Rita B. Ardito, Benedetto Farina, Mauro Adenzato, Giuseppe Carbone, Aurelia Lo Presti, Daniela Palomba, Simone Messerotti Benvenuti

**Affiliations:** a Department of General Psychology, University of Padua, Padua, Italy; b Experimental and Applied Psychology Laboratory, Department of Human Sciences, European University of Rome, Rome, Italy; c Department of Psychology, University of Turin, Turin, Italy; d Padova Neuroscience Center (PNC), University of Padua, Padua, Italy; e Hospital Psychology Unit, Padua University Hospital, Padua, Italy

**Keywords:** Adverse childhood experiences, Anxiety, Depression, ERPs, SPN, P300/LPP

## Abstract

Adverse childhood experiences (ACEs) increase vulnerability to internalizing symptoms, namely symptoms characterized primarily by processes within the self, such as anxiety and depression, but the underlying processes are still unclear. One possible mechanism is that ACEs alter the neural correlates responsible for the preferential processing unpleasant stimuli, a key feature of anxiety. Another mechanism could be a stress-induced disruption in the processing of pleasant stimuli, which is mostly linked with depressive symptoms. In this study, we examined how ACEs and neural correlates of different emotional processing stages (affective engagement, anticipation, elaboration) interact in the association with internalizing symptoms in a sample of university students (*n* = 46, 28 females). Participants completed the Adverse Childhood Experiences Questionnaire (ACE-Q), and the anxiety and depression subscale of the Brief Symptoms Inventory Checklist to assess depression and anxiety. An S1-S2 paradigm, a task in which a cue (S1) anticipates the valence of a succeeding emotional image (S2), was used during an electroencephalographic (EEG) recording. Three event-related potentials (ERPs) reflecting different stages of emotional processing were assessed: the Cue-P300 (reflecting cue-evaluation and affective engagement), the Stimulus Preceding Negativity (SPN; reflecting outcome anticipation), and the P300/late positive potential (LPP) complex (reflecting affective processing). ACEs were linked to greater P300/LPP for unpleasant stimuli, suggesting that childhood adversities may be related to increased elaboration of threatening information. Moreover, ACEs were associated with dampened engagement (Cue-P300) and processing (P300/LPP) of pleasant content. Interestingly, the interaction between the P300/LPP to unpleasant stimuli and ACEs was significantly associated with greater symptoms of anxiety, whereas there was no effect in the link with depression. Specifically, individuals exposed to ACEs only reported heightened anxiety symptoms when their P300/LPP complex to unpleasant stimuli was larger. No significant effect emerged for the other ERPs components. Taken together, these findings suggest that an increased sensitivity to unpleasant content in adulthood might moderate the association between ACEs and anxiety symptoms.

## 1 Introduction

Adverse childhood experiences (ACEs) are highly prevalent, and it is estimated that about one in four children will experience emotional, physical, or sexual abuse in their early lifetime (Sethi et al., 2013; Stoltenborgh et al., 2015). ACEs refer to a wide range of stressful events (e.g., parental maltreatment, life-threatening illness) that occur under the age of 18 and contribute significantly to the global burden of disease (Norman et al., 2012; Massullo et al., 2023). For instance, experiencing ACEs leads to a higher risk of developing physical illness, health risk behaviors, and psychopathology (Fitton et al., 2020; Gilbert, 2012; Norman et al., 2012). Particularly, childhood adversities represent an established vulnerability condition for internalizing disorders - namely symptoms characterized by inner-directed negative emotions, including sadness, loneliness, and worry, which represent a broad range of depression and anxiety symptoms (Krueger and Markon, 2006; Park et al., 2020) - across different age ranges and cultures (Bombay et al., 2011; Cyr et al., 2013; Ferrara et al., 2015; Gilbert et al., 2012; Guo et al., 2021; Jaffee, 2017; Stoltenborgh et al., 2015), and they appear to be responsible for an approximately twofold risk of developing anxiety (Gardner et al., 2019a, Gardner et al., 2019b) and depressive disorders (Gardner et al., 2019a, Gardner et al., 2019b; Green et al., 2010; Moog et al., 2023). Despite the consistent association between early adversities and psychopathology, the mechanisms underlying this association are unclear, nor do we have a complete understanding of the respective potential protective factors (Gerin et al., 2019). However, progress in this area is important to improve targeted psychological interventions (McLaughlin et al., 2019; Oltean et al., 2023). In this context, in line with the diathesis-stress model (Zuckerman, 1999), there may be vulnerability mechanisms that predispose maltreated individuals to the development of internalizing symptoms (Bassani et al., 2013a, Bassani et al., 2013b; McLaughlin et al., 2015; Weinberg, 2023).

One possible mechanism for the link between ACEs and internalizing symptoms is that early adversities and stress may alter the neural mechanisms responsible for processing unpleasant and threatening information, which are key features of anxiety (Pollak and Sinha, 2002; Shackman et al., 2007). This view was first put forward by Pollak in the early 2000s, who suggested that children reared in threatening environments do not passively endure abuse, but instead develop a heightened sensitivity to anger and threat, possibly as a way of anticipating and coping with potential maltreatment (Pollak and Sinha, 2002; Shackman et al., 2007). There is evidence that individuals who have experienced early adversities have increased attention and difficulties in disengaging from unpleasant cues (Hepp et al., 2021; McLaughlin et al., 2015; Pollak and Tolley-Schell, 2003; Shackman et al., 2007). Indeed, a plethora of neuroimaging studies have shown that ACEs are associated with enhanced neural responses to threats in the amygdala and other subcortical structures (e.g., the hippocampus, anterior insula), even in the absence of clinical symptoms (Fonzo et al., 2016; Gerin et al., 2019; Hein and Monk, 2017; Kampa et al., 2024; Saarinen et al., 2021). Moreover, exposure to early adversities has been associated with increased psychophysiological reactivity (i.e., startle reflex, skin conductance) to unpleasant stimuli, even in healthy samples (Jovanovic et al., 2009; Klorman et al., 2003; Koppold et al., 2023; Quevedo et al., 2015). Overall, these patterns of greater behavioral and neural responses to threat are considered important features of internalizing symptoms, particularly in anxiety (Bechor et al., 2019; Cisler and Koster, 2010; Eldar et al., 2010; LeMoult and Gotlib, 2019; Van Bockstaele et al., 2014). While depression is characterized by elevated negative affect, findings on neural responses to unpleasant stimuli remain mixed, with some studies suggesting heightened elaboration (Auerbach et al., 2015; Speed et al., 2016) and others indicating reduced processing (Bylsma, 2021; Foti et al., 2010; Michelini et al., 2021; Weinberg et al., 2016). This latter view aligns with the Emotion Context Insensitivity hypothesis (ECI; Bylsma et al., 2008; Bylsma, 2021; Rottenberg et al., 2005), which suggests that depressive symptoms are associated with a general disengagement with the environment and a reduction in motivated responding to all stimuli (Bylsma et al., 2008; Bylsma, 2021; Rottenberg et al., 2005). Of note, ECI has been confirmed in samples of young adults with depressive symptoms (e.g., Hill et al., 2019; Benau et al., 2019).

Another possible mechanism underlying the association between ACEs and internalizing disorders is the stress-induced disruption of the neural mechanisms responsible for processing pleasant and rewarding stimuli, which are mainly associated with depressive symptoms (Kujawa and Burkhouse, 2017; Pizzagalli, 2014). This view is supported by several studies that reported poorer behavioral performance on reward tasks in individuals with depressive symptoms (e.g., reward learning; Dennison et al., 2019; Hanson et al., 2016). Similarly, in individuals exposed to early adversities, a reduced neural activation to pleasant or rewarding stimuli has been documented (Matz et al., 2010), with some studies indicating decreased activation of several brain regions involved in reward processing, such as the ventral striatum (Goff et al., 2013). However, other studies have found no effect of early adversities on neural responses to pleasant and rewarding stimuli (Boecker-Schlier et al., 2016). Furthermore, the association between early adversities and future depression was enhanced in those who showed reduced neural responses to pleasant and rewarding stimuli (e.g., Burani et al., 2021; Kasparek et al., 2023; Nelson et al., 2016). Interestingly, there is also evidence that in experiments with cue-outcome designs, early adversities are usually associated with reduced anticipation of pleasant cues, rather than their consumption (Hanson et al., 2016; Takiguchi et al., 2015). These previous findings are important because impaired reward processing is associated with anhedonia, a core feature of depression, and may underlie the risk for depression in people who experience early adversities (Pizzagalli, 2014).

Decreased reactivity to positive stimuli, similar to heightened threat sensitivity, may be an adaptive response to an abusive environment. From an evolutionary psychopathology perspective, this reduced motivation for pleasure can be protective by limiting the exploration of dangerous environments and mitigating the pain of abandonment and neglect by caregivers (Del Giudice, 2018, Luyten and Fonagy, 2018). Moreover, negative relational experiences and impairments in appetitive stimuli processing could also result from excessive downregulation of the prefrontal cortex (PFC) through high levels of dopamine in the PFC, which have been observed in response to stress (Pizzagalli, 2014).

Taken together, the reviewed literature indicates that the association between ACEs and internalizing disorders might be moderated by neural responses to emotional stimuli, with the latter acting as a protective or vulnerability factor for the development of anxiety and depressive symptoms in individuals who have been exposed to early adversities. However, some important gaps remain. First, previous studies have considered the impact of ACEs on neural processing of emotional stimuli often neglecting to take into account the differential association with anxiety or depression (Boecker-Schlier et al., 2016; Gerin et al., 2019; Kamkar et al., 2017; Kampa et al., 2024; McLaughlin et al., 2015; Oltean et al., 2023; Saarinen et al., 2021). Moreover, most studies have included individuals who have experienced severe physical and/or sexual abuse, and there is a lack of studies looking at the association between emotional processing and more subtle levels of abuse (e.g., emotional) in determining internalizing symptoms (Sandre et al., 2018). In addition, although ACEs appear to be associated with increased processing of unpleasant stimuli and diminished anticipation and processing of pleasant stimuli, the anticipation and processing of both stimuli categories have not been examined in a single study. Yet, these processes are closely related but distinct at both behavioral and neural levels, each serving unique functions: anticipation prepares individuals for upcoming events, while stimulus response entails immediate engagement with and processing of the affective experience. Hence, it might be useful to clarify the relation between ACEs and distinct phases of affective processing to better understand whether these processes contribute to the risk of internalizing disorders in individuals with greater exposure to ACEs. For instance, examining the anticipation and elaboration of affective stimuli is important as dysfunctions in these stages may play a crucial role in the emergence and maintenance of internalizing disorders in those exposed to adverse rearing environments. Specifically, excessive anticipation of negative experiences may lead to heightened threat sensitivity and chronic hypervigilance, increasing anxiety risk (Sussman et al., 2016), whereas blunted anticipation of positive events may weaken reward responsiveness and motivation, contributing to depression (Olino et al., 2014; Sherdell et al., 2012). In turn, disruptions in stimulus processing - such as prolonged engagement with unpleasant stimuli or diminished processing of pleasant stimuli - may further exacerbate negative affect (Foti et al., 2010; Kujawa et al., 2015; Luking et al., 2016). Together, these altered processes may represent separate key mechanisms linking ACEs to heightened vulnerability for internalizing symptoms.

Furthermore, most of the above-mentioned studies on the processing of appetitive stimuli have relied upon monetary reward tasks, without examining the distinct phases of other appetitive stimuli processing (e.g., pleasant images) that might be more relevant to the context of depression risk (Webb et al., 2021). For example, greater neural responses to pleasant pictures but not monetary rewards have been associated with improved treatment outcomes for depression (Barch et al., 2020). More importantly, due to its poor temporal resolution, fMRI might be inadequate for tracking the distinct phases of appetitive stimulus processing, leading to the merging of neural activity associated with processes that are temporally close but psychologically distinct (i.e., anticipation, and response; Wang et al., 2020).

At the neurophysiological level, event-related potentials (ERPs) are well-suited to distinguish and separate distinct stages of emotional processing due to their excellent temporal resolution (Luck, 2014). Several ERPs reflecting different emotional processing stages and responding to arousing content can be extracted from a two-stimulus task (S1-S2) in which a first stimulus (S1, or cue) indicates the occurrence of the second stimulus (S2, or imperative stimulus) (e.g., Poli et al., 2007). Particularly, in S1-S2 paradigms, two ERPs components are relevant for the study of emotional anticipation, namely the Cue-P300 and the Stimulus Preceding Negativity (SPN) (Buodo et al., 2012; Novak and Foti, 2015; Poli et al., 2007; Simons et al., 1979). The Cue-P300 is a positive deflection occurring approximately 300 ms post-stimulus at parietal sites typically larger for emotional than for neutral cues. It reflects the allocation of attention to the upcoming emotional stimuli (i.e., affective engagement), which then motivates subsequent motivated attention to the S2 cue (Novak and Foti, 2015). The SPN, a negative slow wave that reaches its maximum amplitude approximately 100 ms before the S2, is larger for emotional vs. neutral stimuli and reflects the expectancy and the anticipatory response to these stimuli (Novak and Foti, 2015; Poli et al., 2007). Finally, the P300/late positive potential (LPP) complex is a sustained positive deflection that begins approximately 300–400 ms post-stimulus and reflects the elaborative processing of motivationally salient content, namely motivated attention (Hajcak et al., 2009; Hajcak and Foti, 2020; Moretta and Messerotti Benvenuti, 2023; Schupp et al., 2000). The initial portion of this ERP (P300) is similar in morphology and timing to the P300 observed in cognitive tasks, such as oddball paradigms, and is sensitive to emotional stimuli (e.g., Weinberg et al., 2015). According to Hajcak and Foti (2020), the P300/LPP complex reflects output from a common neural system that responds to stimulus significance, whether task-related, emotionally evocative, or individualized. Indeed, an increased P300/LPP complex has been consistently reported in response to emotional stimuli compared to neutral stimuli (Palomba et al., 1997; Hajcak and Foti, 2020; Schupp et al., 2000).

Overall, neural responses to emotional stimuli might be a moderator of the association between ACEs and internalizing symptoms, as both maltreated individuals and those with internalizing disorders show similar patterns of emotional processing assessed by ERPs. Preliminary evidence suggests that individuals exposed to early adversities and stress may show an enhanced P300/LPP complex in response to unpleasant stimuli (Hedrick et al., 2024; Sandre et al., 2018; Shackman et al., 2007), and a blunted P300/LPP complex in response to pleasant stimuli (Pollak et al., 1997). Thus, these earlier ERPs studies suggest that individuals exposed to ACEs may be particularly sensitive to unpleasant stimuli and less sensitive to pleasant stimuli. Consistently, some studies indicate an enhanced P300/LPP complex for unpleasant stimuli in anxiety (Bylsma et al., 2022; Huang et al., 2023; MacNamara and Hajcak, 2010; Pedersen and Larson, 2016), and the limited studies examining anticipatory ERPs suggest heightened anticipation of unpleasant stimuli in anxiety (SPN; Huang et al., 2023). Regarding depression, diminished anticipation (Cue-P300, SPN; Novak et al., 2016; Umemoto and Holroyd, 2017) and elaboration (P300/LPP complex; Klawohn et al., 2021; Bylsma, 2021; Dell'Acqua et al., 2022b; Luckhardt et al., 2023; MacNamara et al., 2016; Moretta et al., 2021; Weinberg et al., 2016) of pleasant stimuli has been documented in samples with depressive symptoms.

This study aimed to improve understanding of the effects of exposure to ACEs on emotional anticipation and processing in a sample of healthy young adults with varying levels of anxiety and depressive symptoms. Specifically, the hypotheses were the following:

I) Exposure to ACEs would be associated with a) enhanced processing of unpleasant stimuli, indexed by greater P300/LPP complex amplitude for unpleasant images compared to neutral images, and b) reduced processing of pleasant stimuli, indexed by blunted P300/LPP complex amplitude for pleasant images compared to neutral images.
II) Exposure to ACEs would be associated with higher anxiety and depressive symptoms. In addition, it was predicted that the P300/LPP complex would moderate this association, such that a) participants with a larger P300/LPP complex to unpleasant stimuli would show a stronger association between ACEs and anxiety symptoms than participants with a smaller P300/LPP complex, and b) participants with a smaller P300/LPP complex to pleasant stimuli would show a stronger association between ACEs and depressive symptoms than participants with a larger P300/LPP complex.

Given the lack of previous research examining anticipatory ERPs (Cue-P300 and SPN) and their association with ACEs, this study was exploratory. Therefore, no pre-defined hypotheses were made regarding their relationship with ACEs or their potential impact on anxiety and depressive symptoms.

## 2 Methods

A total of 46 (28 females, mean age = 22.93, standard deviation = 3.37, range = 19–35) Italian Caucasian students from the University of Padua (Italy) voluntarily participated in the study. An a priori power analysis was performed using G∗Power 3.1 software (Faul et al., 2009). It revealed that at a probability level of 0.05, a sample size of 46 was required to achieve a satisfactory statistical power (1–β = 80 %) with an effect size of f2 = 0.35 in a two-sided test linear regression model with 6 total number of predictors (see statistical analysis section). This effect size was estimated following a previous study (Sandre et al., 2018) investigating the association between ERP data (i.e., LPP), childhood maltreatment, and depressive and anxiety symptoms (ηp2 = 0.26, f^2^ conversion = 0.35; Cohen, 2013; Kim, 2016). The enrolled sample was medically healthy and free of psychotropic medication, as assessed with an ad-hoc anamnestic interview. Exclusion criteria included a current and past history of psychiatric and neurological diseases. All participants had normal or corrected-to-normal vision and were unaware of the purpose of the experiment. All participants read, understood, and signed the informed consent. The study was conducted in compliance with the World Medical Association Declaration of Helsinki on research involving human subjects and was approved by the Ethical Committee of Psychological Research, Area 17, University of Padova (prot. 220-c). Participants received no remuneration or other compensation (i.e., academic credit).

### 2.1 Measures

#### 2.1.1 Psychological measures

Anxiety and depressive subscales of the Brief Symptom Inventory (BSI) (Derogatis and Melisaratos, 1983; Italian version, De Leo et al., 1993) were used to assess the levels of such symptomatology. Each subscale consists of six items that evaluate typical symptoms of anxiety (e.g., nervousness, tension) and depression (e.g., loss of interest, loneliness, suicide ideation).

Items are scored on a 5-point Likert scale ranging from 0 (“*not at all*”) to 4 (“*extremely*”) with higher score reflecting higher depressive and anxiety symptoms. Both subscales are characterized by satisfactory psychometric properties (Khalil et al., 2011) and are considered suitable tools for the assessment of these psychiatric symptoms (Alshogran et al., 2022; Riskind et al., 2014).

Early adversities were assessed using the 10-item version of the Adverse Childhood Experiences Questionnaire (ACE-Q, Felitti et al., 1998). The ACE-Q is a retrospective self-report measure that comprises 10 yes/no items assessing exposure to physical, emotional, and sexual abuse, and household dysfunction (witnessing domestic violence, substance abuse, mental illness, and incarceration). Higher scores indicate a higher number of potentially traumatic events in childhood.

#### 2.1.2 Experimental task

EEG was recorded while participants underwent an S1-S2 affective paradigm. The paradigm included a total of 72 trials, each starting with a 500 ms baseline (a white fixation dot), followed by a symbol cue (S1) lasting 1500 ms that signaled the emotional valence (pleasant, neutral, unpleasant) of the upcoming picture (S2), which was presented 4500 ms later and lasting 2000 ms. The S2 was followed by a variable intertrial interval (ITI) of 3000–4000 ms, during which a white fixation dot (identical to the baseline) was presented. The S1 cue was a symbol that consistently signaled the valence of the upcoming S2 image: a "+" indicated pleasant, a "–" indicated unpleasant, and a white circle indicated neutral content. Participants were asked to attend to the cue (S1) and the subsequent image (S2). A motor response to the second stimulus was not required. The S2 comprised 72 images (600 × 800 pixels), divided into three categories: 24 pleasant (e.g., erotic couples, sports), 24 neutral (e.g., neutral faces, household objects), and 24 unpleasant (e.g., attacking humans and animals) selected from the International Affective Picture System (IAPS; Lang et al., 1997) based on their standardized valence and arousal ratings (see Moretta et al., 2021). Pictures were presented in a semi-randomized sequence (i.e., no more than two S1-S2 pairs of the same emotional valence were shown consecutively). Only highly arousing pleasant and unpleasant pictures were selected, as these have been shown to induce elevated psychophysiological changes (e.g., Bradley et al., 2001). Pleasant and unpleasant pictures were matched for normative arousal ratings (unpleasant, mean ± SD = 6.5 ± 0.5; pleasant, mean ± SD = 6.5 ± 0.4; *p* = .92), which were significantly higher than for neutral pictures (neutral, mean ± SD = 2.9 ± 0.7; all *p*s < 0.001).^1^ At the end of the task, 36 pictures (12 for each emotional valence) were presented again in order to obtain the subjective ratings of emotional valence and arousal using the 9-point Valence and Arousal scales of the Self-Assessment Manikin (SAM; Bradley and Lang, 1994).

### 2.2 Procedure

Students from the University of Padua completed an online survey (via Google Modules) to assess the inclusion criteria for the study, and then completed the BSI and ACE-Q questionnaires. An appointment was then made for a laboratory session at the Department of General Psychology of the University of Padua. Before the experimental session, participants were asked not to drink alcohol the day before and to abstain from caffeine and nicotine on the day of the appointment. Upon arrival at the laboratory, after participants had read and signed the written informed consent form, they were administered the ad-hoc anamnestic interview. Participants were then seated on a comfortable chair in a dimly lit, sound-attenuated room. After electrode attachment and a 3-min resting-state period, three practice trials were conducted, including one pleasant, one neutral, and one unpleasant trial. The participants then underwent the S1-S2 paradigm. The entire procedure took about 90 min.

### 2.3 Electroencephalogram data acquisition and analysis

EEG was recorded using a 32-channel ANT system and a computer running eego™ software (ANT Neuro, Enschede, Netherlands). The elastic cap with 32 tin electrodes was arranged according to the 10–20 System (Fp1, Fpz, Fp2, F7, F3, Fz, F4, F8, FC5, FC1, FC2, FC6, T7, C3, Cz, C4, T8, CP5, CP1, CP2, CP6, P7, P3, Pz, P4, P8, POz, O1, Oz, O2, and M1 and M2 [mastoids]), referenced online to CPz. To monitor eye movements and blinks, vertical and horizontal electrooculograms (EOGs) were recorded using a bipolar montage. Electrode impedance was kept below 10 kΩ. The EEG signal was sampled at 1000 Hz with filter settings from DC to 30 Hz.

**Fig. 1:**
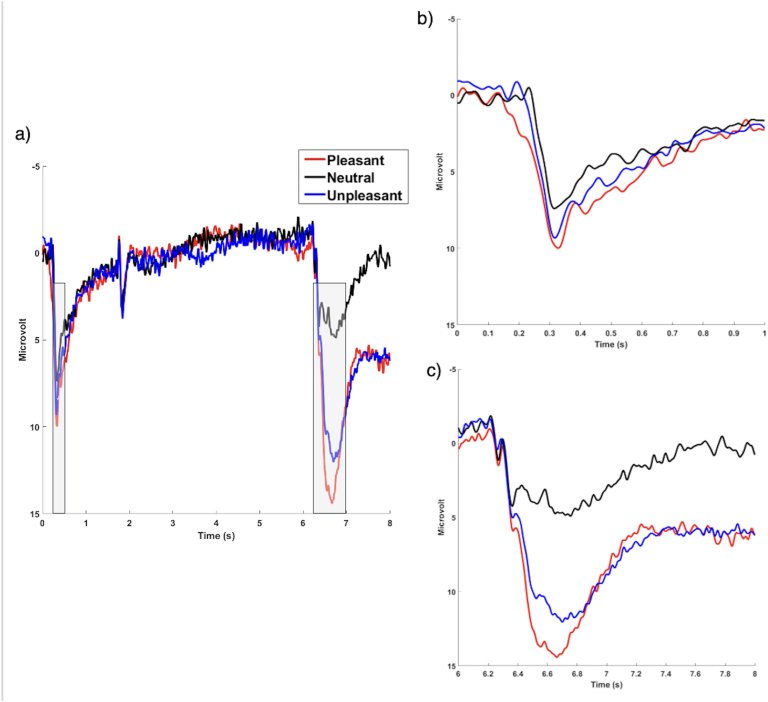
Grand average ERP waveforms during the S1-S2 task presented for the average of parietal electrodes (P3, PZ, P4). Cue onset was at 0 s, and image onset was at 6 s. The Cue-P300 was scored as the peak amplitude in the first shaded window (0.2–0.4 s, Panel a). The P300/LPP complex was scored as the peak amplitude in the second shaded window (6.3–7 s, Panel b).

**Fig. 2:**
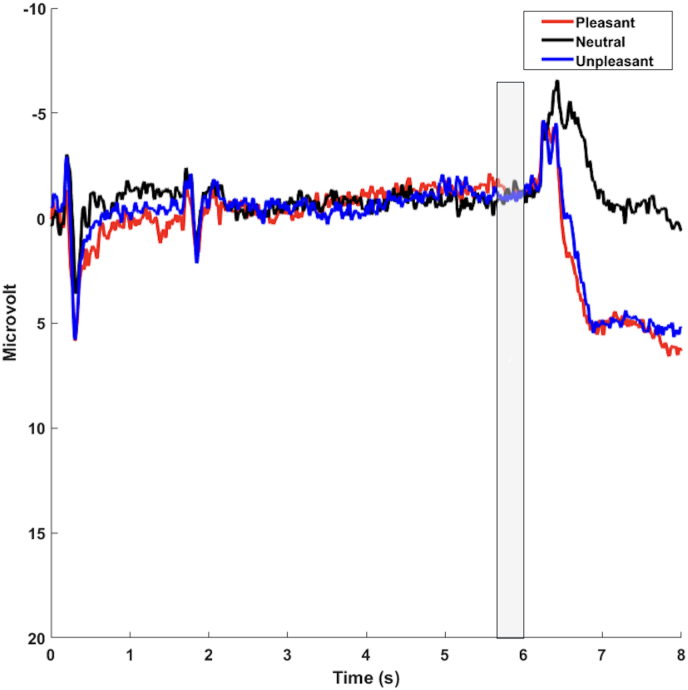
Grand average ERP waveforms during the S1-S2 task, presented for the average of the frontal electrodes (F3, FZ, F4). Cue onset was at 0 s, and image onset (S2) was at 6 s. The SPN was scored as the average activity in the shaded window (5.8–6 s).

The EEG signal was downsampled to 500 Hz and re-referenced offline to the linked mastoids as implemented in EEGLAB (Delorme and Makeig, 2004). Further analysis was performed using Brainstorm (Tadel et al., 2011). Data were band-pass filtered from 0.01 to 30 Hz and corrected for blink artifacts using independent component analysis (ICA). Then EEG signal was epoched into 8250 ms segments (from 250 ms before S1 until 2000 ms after S2 onset, i.e., −250 to 8000 ms). The signal was then baseline-corrected from -250 -50 ms before S1. A semiautomatic procedure was employed to detect and reject artifacts. The criteria applied was a voltage difference of 200 μV. Visual inspection of the data was then conducted to detect and reject any remaining artifacts. The ERP time windows and electrodes were chosen based on topographical visualization of the grand average of the three categories and to be consistent with previous studies. Particularly, visual inspection of grand averages across all participants in the three emotional categories confirmed that the Cue-P300 and the P300/LPP complex were maximal at parietal sites, consistent with previous research (Dell'Acqua et al., 2022a, Dell'Acqua et al., 2022b; Moretta and Messerotti Benvenuti, 2023; Novak et al., 2016; Novak and Foti, 2015; Poli et al., 2007; Palomba et al., 1997; Schupp et al., 2000). Therefore, the Cue-P300 and the P300/LPP complex (Fig. 1) were scored by averaging peak amplitudes at electrodes P3, Pz, and P4, from 200 to 400 ms post-S1 and 300–1000 ms post-S2, respectively. Peak amplitude measurement is standard for P300-like components because it captures the maximal engagement during this early evaluative stage (Polich, 2007; Kok, 2001). In agreement with previous research (e.g., Buodo et al., 2012; Poli et al., 2007), the SPN was scored as the mean amplitude in the 200 ms preceding the image onset at electrode sites at frontal sites (F3, FZ, F4, Fig. 2). Grand averages of single frontal (F3, FZ, F4), central (C3, CZ, C4), and parietal (P3, PZ, P4) electrodes are provided in the supplementary material (Fig. S1).

### 2.4 Statistical analyses

The statistical analyses were performed in Rstudio (Rcore team, 2023). A *p*-value of 0.05 was the cut-off value for statistical significance. First, descriptive statistics and Pearson correlations between BSI scales and ACE-Q were conducted.

Repeated measures analyses of variance (ANOVAs) with Valence (pleasant, neutral, unpleasant) as a within-subjects factor were performed for self-reported valence and arousal.

Considering that from the visual inspection of grand averages, the SPN did not show the expected pattern of larger negativity to emotional vs. neutral, a linear mixed-effects model for the SPN mean amplitude was performed to clarify whether there was an effect of emotional valence on this ERP using the *lme4* (Bates et al., 2015) and *lmerTest* (Kuznetsova et al., 2017) packages. The model included the participant as a random intercept, while the Valence (pleasant, neutral, unpleasant) was specified as a fixed factor: *Model ← lmer(SPN amplitude ∼ Valence + (1|Subject))*. The *p*-values obtained by the Satterthwaite approximation (implemented in the *lmerTest* library) were reported.

To test hypothesis (I), namely, to explore the association between ACEs and emotional processing stages for emotional vs. neutral images, two repeated measures linear mixed-effects models were conducted for each ERP. All models included the participant as a random intercept, while the Valence (pleasant, neutral, unpleasant) and ACEs, and their interaction were specified as fixed factors: *Model ← lmer(ERP amplitude ∼ Valence × ACE-Q scores + (1|Subject))*. For the fixed effects, the estimated coefficient (*b*), standard error (SE), *t* values, and confidence intervals were reported for each parameter included in the final model. In addition, the *p*-values obtained by the Satterthwaite approximation (implemented in the lmerTest library) were reported.

To test hypothesis (II), four linear regression models were conducted to separately assess the main effect of ACEs and ERPs amplitude for affective images (differential scores of pleasant and unpleasant) and their interaction in determining anxiety and depressive symptoms. To reduce the number of predictors, differential scores for ERPs (pleasant – neutral, unpleasant – neutral) were used. In detail, the models were specified as follows: *Model ← lm(BSI Anxiety or BSI depression scores ∼* △*ERPs to unpleasant × ACE-Q scores +* △*ERPs to pleasant × ACE-Q scores).*

All predictors were centered and scaled: the mean of each variable was subtracted from each value and the resulting value was then divided by the standard deviation of its distribution. Collinearity was tested by calculating the Variance Inflation Factors (VIF) using the *vif* function of the car package (Fox et al., 2019). For significant categorical main effects (*p* < .05), Tukey HSD post-hoc tests were performed to correct for multiple comparisons.

## 3 Results

### 3.1 Psychological measures and valence and arousal self-report ratings

The average BSI anxiety score was 6.80 (SD = 4.72, range = 1–17) and the average BSI depression score was 8.31 (SD = 5.67, range 1–21). Further, the ACE-Q score average was 1.33 (SD = 1.33, range = 0–7). Correlations among study variables are shown in Table 1.

**Table 1:** Pearson correlations among study variables.

	ACE-Q	BSI Anxiety	BSI Depression	P300/LPP pleasant (differential score)	P300/LPP unpleasant (differential score)
**ACE-Q**	–				
**BSI Anxiety**	0.16	–			
**BSI Depression**	0.10	0.68∗	–		
**P300/LPP pleasant (differential score)**	−0.20	−0.32∗	−0.27^§^	–	
**P300/LPP unpleasant (differential score)**	0.21	0.01	−0.17	0.33∗	–

Note. ∗p < .05, §p = .07.

The ANOVA on valence ratings yielded a significant main effect for Valence, F_(2,88)_ = 144.00*, p* < .00.001, η2p = .77. Unpleasant pictures were evaluated as significantly more unpleasant than neutral (*p*_Tukey_ < 0.001) and pleasant (*p*_Tukey_ < 0.001) pictures. Pleasant stimuli were rated as significantly more pleasant than neutral ones (*p*_Tukey_ < 0.001). The ANOVA on arousal ratings revealed a significant main effect for Valence, F_(2,88)_ = 155.00*, p* < .00.001, η2p = .78. Specifically, arousal ratings were higher for both pleasant and unpleasant pictures compared to neutral ones (all *p*s_Tukey_ < 0.001). Unpleasant pictures were rated as more arousing than pleasant stimuli (*p*_Tukey_ = 0.04). Table 2 shows the means and standard deviation of SAM valence and arousal ratings in this sample.

**Table 2:** Mean and standard deviation of self-reported SAM (self-assessment manikin) valence and arousal ratings.

	Valence	Arousal
Pleasant	6.68 ± 1.01	5.00 ± 1.51
Neutral	5.14 ± 0.76	2.25 ± 1.24
Unpleasant	3.02 ± 1.19	5.41 ± 1.80

### 3.2 SPN results

The results of the mixed-effect models determining the SPN mean amplitude from Valence (pleasant, neutral, unpleasant) did not yield a significant effect of Valence (all *p*s > 0.53, pleasant = −0.54 ± 5.40 μV, neutral = −0.84 ± 5.68 μV, unpleasant = −0.30 ± 6.35 μV), supporting that, as evident from the visual inspection of the grand averages, the SPN did not present the expected pattern of larger negativity to emotional vs. neutral stimuli. Hence, the SPN could not be considered for further statistical analyses in this sample.

### 3.3 The influence of ACEs on emotional processing

The results of the mixed-effect models determining ERPs (Cue-P300, P300/LPP complex) from ACE-Q scores are shown in Table 3.

**Table 3:** Estimated parameters of the linear mixed-effects examining associations between ERPs (Cue-P300, P300/LPP complex) and Valence, ACE-Q scores and their interaction. The baseline is Neutral trials. Significant effects are shown in bold.

Predictor	*b* (SE)	*t*	*p*
**Cue-P300 model**			
**Valence – pleasant**	**2.50 (0.35)**	**7.10**	**< 0.001**
**Valence – unpleasant**	**1.63 (0.35)**	**4.61**	**< 0.001**
ACE-Q scores	0.34 (0.62)	0.56	0.58
Valence pleasant × ACE-Q scores	−0.85 (0.36)	−2.39	0.02
Valence unpleasant × ACE-Q scores	−0.24 (0.36)	−0.67	0.50
**P300/LPP complex model**			
**Valence – pleasant**	**7.53 (0.68)**	**11.11**	**< 0.001**
**Valence – unpleasant**	**4.74 (0.68)**	7.00	**< 0.001**
ACE-Q scores	0.36 (0.90)	0.40	0.69
Valence pleasant × ACE-Q scores	−1.26 (0.68)	−1.85	0.06
**Valence unpleasant × ACE-Q scores**	**1.36 (0.68)**	**2.00**	**0.04**

Note. SE = standard error; ACE-Q = score of the Adverse Childhood Experiences questionnaire.

**Fig. 3:**
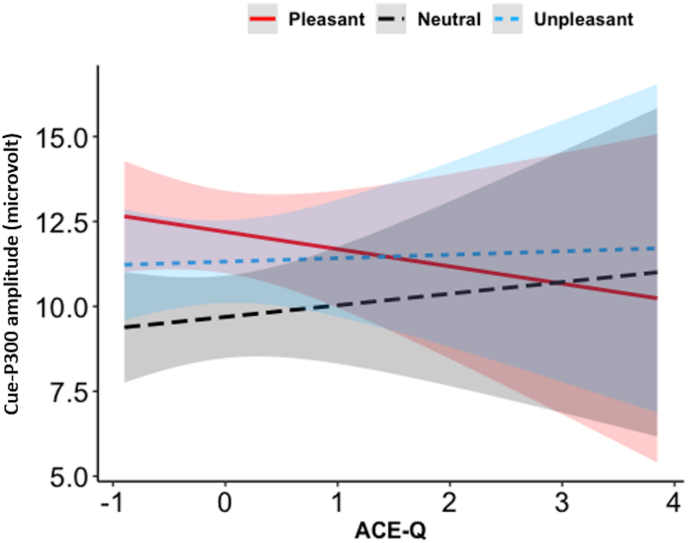
Interaction effect of ACE-Q scores (mean-centered values) and Valence on the Cue-P300 amplitude (microvolts). Ninety-five % confidence bands are presented in different colors. ACE-Q = score of the Adverse Childhood Experiences questionnaire.

In the Cue-P300 model, there was a significant effect of Valence, such that the amplitude was larger for both pleasant and unpleasant relative to neutral trials (pleasant vs. neutral, *p*_*Tukey*_ <0.0001, unpleasant vs. neutral, *p*_*Tukey*_ <0.0001; peak amplitude to pleasant = 12.18 ± 5.63 μV, neutral = 9.67 ± 4.18 μV, unpleasant = 11.30 ± 4.37 μV). Pleasant trials showed larger amplitude than unpleasant ones (*p*_*Tukey*_ = 0.34). Also, a significant interaction between ACE-Q and Valence emerged, such that greater ACE-Q scores were associated with smaller Cue-P300 amplitude to pleasant trials (Fig. 3).

**Fig. 4:**
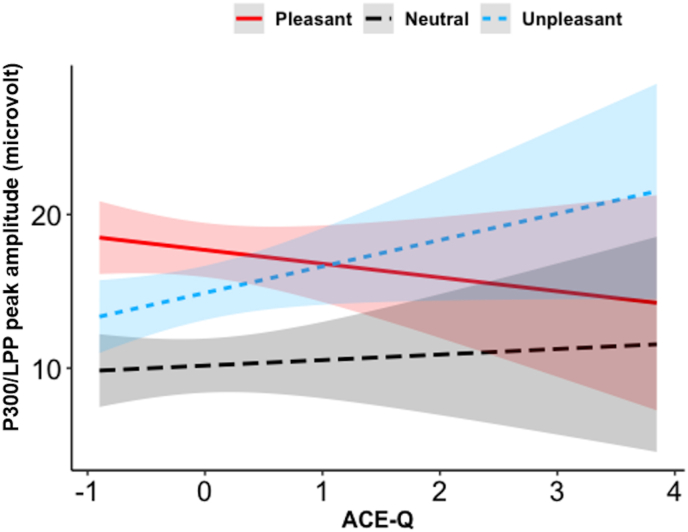
Interaction effect of ACE-Q scores (mean-centered values) and Valence on P300/LPP complex amplitudes (microvolts). Ninety-five % confidence bands are presented in different colors. ACE-Q = score of the Adverse Childhood Experiences questionnaire.

In the P300/LPP complex model, the effect of Valence was significant, namely pleasant, and unpleasant images elicited a larger amplitude relative to neutral ones (pleasant vs. neutral, *p*_*Tukey*_ <0.0001, unpleasant vs. neutral, *p*_*Tukey*_ <0.0001; peak amplitude to pleasant = 17.80 ± 8.11 μV, neutral = 10.16 ± 6.71 μV, unpleasant = 14.90 ± 7.99 μV). Moreover, Tukey HSD post-hoc tests revealed pleasant images elicited a larger P300/LPP complex relative to unpleasant ones (*p*_*Tukey*_ < 0.0001). Further, a significant interaction between ACE-Q scores and Valence emerged, such that individuals with higher ACE-Q scores had an increased P300/LPP complex amplitude to unpleasant and a marginally significant decreased P300/LPP complex amplitude to pleasant images (Fig. 4). VIF values were all <1.50, suggesting adequate levels of multicollinearity among the predictor variables.

### 3.4 The moderating role of emotional processing in the association between ACEs and anxiety and depressive symptoms

**Fig. 5:**
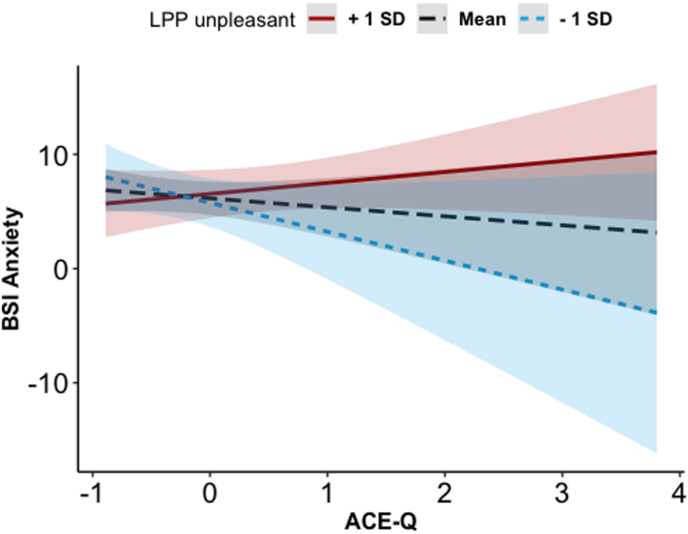
Interaction effect of ACE-Q (mean-centered values) and △P300/LPP complex to unpleasant pictures (in microvolts) in determining anxiety symptoms. Ninety-five % confidence bands are presented in different colors. ACE-Q = score of the Adverse Childhood Experiences questionnaire.; BSI = Brief Symptom Inventory.

The results of the linear models determining anxiety and depressive symptoms from the ERPs (Cue-P300 and P300/LPP complex), the ACE-Q scores, and their interactions are shown in Table 4, Table 5, respectively. As for the model determining anxiety from the Cue-P300, there was no significant effect. Instead, the P300/LPP complex model showed a significant effect of P300/LPP complex amplitude on pleasant images, such that individuals with smaller amplitudes had higher anxiety symptoms. Moreover, there was a significant interaction between ACE-Q scores and the P300/LPP complex to unpleasant images, such that higher ACE-Q scores were linked with more anxiety symptoms when the P300/LPP amplitude was larger on unpleasant images (Fig. 5).^2^ Finally, the models determining depressive symptoms using ERPs and ACE-Q scores showed no significant effect (Table 5). The VIF values were all <2.74, suggesting adequate levels of multicollinearity among the predictor variables.

**Table 4:** Linear regression model examining the main and interactive association between ACE-Q scores and ERPs to pleasant and unpleasant stimuli with anxiety symptoms.

Predictor	*b* (SE)	*t*	*P*
**Model predicting anxiety symptoms from the Cue-P300**			
△Cue-P300 to pleasant images	−0.33 (0.87)	−0.38	0.71
△Cue-P300 to unpleasant images	−1.30 (0.86)	−1.51	0.14
ACE-Q scores	1.10 (0.83)	1.28	0.21
△Cue-P300 to pleasant images × ACE-Q scores	1.37 (1.50)	0.91	0.37
△Cue-P300 to unpleasant images × ACE-Q scores	−0.12 (0.84)	−0.14	0.89
**Model predicting anxiety symptoms from P300/LPP complex**			
△**P300/LPP complex to pleasant images**	**−1.91 (0.75)**	**−2.56**	**0.01**
△P300/LPP complex to unpleasant images	0.40 (0.75)	0.52	0.61
ACE-Q scores	−0.79 (0.87)	−0.90	0.37
△P300/LPP complex to pleasant images × ACE-Q scores	−1.42 (0.91)	−1.56	0.13
△**P300/LPP complex to unpleasant images × ACE-Q scores**	**1.74 (0.84)**	**2.10**	**0.04**

*Note. b = unstandardized coefficient; SE = standard error;* ACE-Q = *score of the Adverse Childhood Experiences questionnaire.* △ = differential score.

**Table 5:** Linear regression model testing the main and interactive association between ACEs and ERPs to pleasant and unpleasant stimuli with depressive symptoms.

Predictor	B (SE)	*t*	*p*
**Model predicting depressive symptoms from Cue-P300**			
△Cue-P300 to pleasant images	−0.31 (1.05)	−0.30	0.77
△Cue-P300 to unpleasant images	−1.39 (1.04)	−1.37	0.20
ACE-Q scores	1.16 (1.01)	1.15	0.26
△Cue-P300 to pleasant images × ACE-Q scores	1.95 (1.81)	1.08	0.29
△Cue-P300 to unpleasant images × ACE-Q scores	0.34 (1.10)	0.34	0.73
**Model predicting depressive symptoms from P300/LPP complex**			
△P300/LPP complex to pleasant images	−1.42 (0.95)	−1.50	0.14
△P300/LPP complex to unpleasant images	−0.70 (0.96)	−0.68	0.50
ACE-Q scores	−0.34 (1.12)	−0.31	0.76
△P300/LPP complex to pleasant images × ACE-Q scores	−1.10 (1.17)	−0.94	0.35
△P300/LPP complex to unpleasant images × ACE-Q scores	1.02 (1.10)	0.95	0.35

*Note. b = unstandardized coefficient; SE = standard error; ACE-Q = score of the Adverse Childhood Experiences questionnaire.* △ = differential score.

## 4 Discussion

Exposure to adverse childhood experiences is a vulnerability factor for the development of anxiety and depressive symptoms. Identifying potential moderators that increase or decrease this association is important for early detection and targeted clinical intervention following maltreatment. Therefore, this study aimed to improve the understanding of how exposure to ACEs influences emotional anticipation and processing, and how the interaction between these factors influences the presence of anxiety and depressive symptoms. The results showed that individuals who had experienced more adversities were more likely to have a heightened P300/LPP complex for unpleasant stimuli, a reduced Cue-P300, and a marginally significant P300/LPP complex for pleasant stimuli. Additionally, individuals with more ACEs exhibited higher anxiety symptoms only when their P300/LPP complex to unpleasant images was heightened, while those with more ACEs and a lower P300/LPP complex reported lower anxiety symptoms. Finally, ACEs did not interact with emotional processing in determining depressive symptoms.

Consistent with previous studies showing that individuals exposed to ACEs tend to show heightened neural responses to unpleasant stimuli (Fonzo et al., 2016; Gerin et al., 2019; Hedrick et al., 2024; Matz et al., 2010; Sandre et al., 2018), the results indicated that individuals with greater ACEs tended to show higher amplitudes of the P300/LPP complex in response to unpleasant stimuli. Even minor emotional and physical abuse, as assessed by the ACE-Q, appears to potentially increase the responsiveness of brain regions associated with threat recognition. This may make some individuals more prone to recognizing and focusing on unpleasant cues in their environment, possibly as an adaptive response to challenging social environments such as dysfunctional households. While this heightened vigilance may be beneficial for navigating threatening environments, particularly in children (Klorman et al., 2003; Pollak et al., 1997), it may also contribute to the development of maladaptive behaviors such as excessive worry, hypervigilance, or avoidance. Furthermore, as expected from the literature, exposure to greater ACEs was associated with an attenuated Cue-P300 and P300/LPP complex to pleasant stimuli. This is consistent with animal and human models suggesting that prolonged exposure to stress, particularly during development, negatively impacts the neural mechanisms responsible for anticipating and processing pleasant/rewarding stimuli (e.g., Dillon et al., 2009; Pizzagalli, 2014). One possible explanation for the finding that ACEs were associated only with a blunted Cue-P300 response to pleasant cues—and not with any effect on unpleasant cues—is the presence of an overall valence effect in the sample. Specifically, the entire sample showed greater affective engagement with pleasant cues than with unpleasant ones, suggesting that positive stimuli elicited more robust Cue-P300 across participants. This general bias toward pleasant cues may have reduced the likelihood of detecting associations with unpleasant stimuli in this phase of affective processing.

As hypothesized, the analysis of the interaction between affective processing and ACEs in determining anxiety symptoms showed a significant interaction. Individuals exposed to ACEs reported heightened anxiety symptoms only when their P300/LPP complex to unpleasant stimuli was larger. To our knowledge, this is the first study showing a unique moderating role of the P300/LPP complex to unpleasant stimuli in the association between ACEs and anxiety symptoms. Moreover, this aligns with an existing framework suggesting that ACEs heighten the likelihood of anxiety, as some individuals raised in stressful and unpredictable settings may not develop the skills to recognize and appropriately interpret emotional content (Poole et al., 2018). Conversely, other studies have not confirmed or investigated this association. For example, Sandre and colleagues (2018) found no significant associations between the magnitude of the P300/LPP complex and anxiety in a sample of young adults who had experienced emotional and/or physical abuse. Instead, the study suggests that increased unpleasant affective processing may represent a latent vulnerability factor for future symptoms (Sandre et al., 2018). These discrepancies could be explained by the presence of additional protective or vulnerability factors underlying the association between ACEs, the P300/LPP complex, and current anxiety symptoms that were not considered. For example, it would be interesting to investigate the role of social support in protecting maltreated individuals with heightened P300/LPP complex from developing anxiety in response to unpleasant stimuli (Auerbach et al., 2011; Ditzen and Heinrichs, 2014; Taylor, 2011; Turner and Brown, 2010). Moreover, higher ACE scores were associated with lower anxiety when P300/LPP to unpleasant stimuli was reduced, suggesting that less elaboration of unpleasant stimuli may serve as a protective mechanism against anxiety in those exposed to greater adverse childhood experiences.

Interestingly, anxiety symptoms were also determined by the P300/LPP complex to pleasant stimuli. Although unexpected, a similar association has been previously reported (Dell'Acqua et al., 2022a, Dell'Acqua et al., 2022b). This could be explained by the fact that selective and sustained processing of unpleasant stimuli, a core feature of anxiety symptoms (e.g., Weinberg et al., 2016), could lead to reduced processing of other salient or relevant information in the environment (i.e., pleasant content). However, as few studies have included positively valenced stimuli when assessing the P300/LPP complex in individuals with anxiety symptoms, the processing of pleasant information in anxiety remains unclear.

With regard to depression, neither affective engagement (as indexed by the Cue-P300) nor motivated elaboration (as reflected by the P300/LPP responses to pleasant stimuli) moderated the association between ACEs and depressive symptoms, contrary to our hypotheses. Nonetheless, it could be speculated that the observed blunted Cue-P300 to pleasant cues in individuals with greater ACEs might represent a vulnerability factor for the development of depressive symptoms, consistent with prior findings linking this pattern with depressive symptomatology (e.g., Thompson et al., 2023 ). These null findings could be explained by the fact that dampened affective engagement (Cue-P300) and emotional processing (P300/LPP) of pleasant stimuli may be more closely associated with a dimension of anhedonia (i.e., loss of pleasure/lack of reactivity to appetitive stimuli), a specific depressive symptom known to be closely related to reward system function (Barch et al., 2020; Pizzagalli, 2014) and which was not measured in the current study. Moreover, while mild exposure to ACEs may heighten threat sensitivity, leading to exaggerated processing of negative stimuli, blunted appetitive processing, and, consequently, depressive symptoms, might emerge only after prolonged or severe adversity. Hence, prolonged exposure to stress and/or more severe ACEs could contribute to a broader dysregulation of affective processing, including blunted responses to pleasant stimuli, which is more strongly associated with depression. However, this hypothesis could not be directly tested in the present study, as ACE scores in the sample were relatively low.

Moreover, the interaction between ACEs and P300/LPP to unpleasant stimuli was not linked with depressive symptoms. This aligns with the inconsistency of findings on the processing of unpleasant stimuli in depression, with some studies reporting heightened elaboration (e.g., Burkhouse et al., 2017) and others indicating a blunted response (Foti et al., 2010; Weinberg et al., 2016), in agreement with the ECI model (Rottenberg et al., 2005; Bylsma et al., 2008; Bylsma, 2021). Accordingly, the results of the current study provide further support for competing theories regarding emotional responses across the dimensions of anxiety and depression (Hill et al., 2019; Weinberg et al., 2016). Specifically, the present data suggest that anxiety is predominantly associated with heightened sensitivity to threat, whereas depression does not show the same pattern. Thus, examining emotional response patterns—particularly the motivated elaboration of stimuli—may aid in distinguishing emotional processing profiles that are more closely linked to anxiety symptoms and their trajectory, especially in threat-related contexts.

In the current sample, the SPN did not show the expected pattern of larger amplitude in the anticipation of emotional pictures compared to neutral pictures across the sample. Hence, it could be that the paradigm used did not sufficiently activate the anticipation mechanisms of the upcoming stimuli or that the stimuli were not appealing enough for the sample. Another task feature that may have led to this null result is the fact that in this study the valence of the S1 always matched the valence of the S2, meaning that participants were able to predict the nature of the upcoming stimulus with a high degree of certainty. This design choice was made to maintain consistency with previous studies (e.g., Buodo et al., 2012; Poli et al., 2007; Takeuchi et al., 2005). However, it is important to note that other studies have manipulated the congruency between S1 and S2 (e.g., Del Popolo Cristaldi et al., 2022; Del Popolo Cristaldi et al., 2022), which reduces predictability. Such variations in task design may enhance the engagement of anticipatory mechanisms by requiring participants to be more vigilant, potentially leading to a more pronounced SPN response. Future research should consider incorporating such manipulations to better understand the dynamics of anticipation and emotional processing.

Moreover, on the subjective level, the self-report rating of valence and arousal confirmed that the experimental manipulation was effective. In addition, unpleasant pictures were rated as more arousing than pleasant ones. This is in line with other studies that reported similar results (Dell'Acqua et al., 2022a, Dell'Acqua et al., 2022b; Messerotti Benvenuti et al., 2020, Weinberg and Hajcak, 2010). Considering that the IAPS pictures were selected because they showed the same arousal ratings in the initial validation (Lang et al., 1997), these results may indicate that a newer validation of the IAPS stimuli is needed. However, the larger P300/LPP peak amplitude in response to pleasant and unpleasant compared to neutral stimuli demonstrates a neural modulation to highly arousing images compared to low arousing images Lang and Bradley, 2010, further confirming the efficacy of the experimental manipulation.

The results of this study appear to be in line with promising research on endophenotypes of the main psychopathological dimensions of anxiety and depression. An endophenotype is a biological trait that is assumed to be involved in the causal pathway from genes to the clinical manifestation of the disorder (Gottesman and Gould, 2003, Lenzenweger, 2013). From a clinical standpoint, the study of endophenotypes in psychopathology appears to be a promising avenue for understanding the intricate interweaving of risk factors, causes and pathogenetic processes that cause and maintain mental disorders, and would facilitate their prevention and the early identification of the most effective treatments.

Moreover, the present study has added new insights to the literature aimed at developing empirically based screening, prevention, and intervention programs for children and young adults who have been emotionally and/or physically abused. For example, maltreated individuals with a heightened P300/LPP complex to unpleasant stimuli may be more vulnerable to the development of anxiety and may require more targeted interventions. Therefore, integrating a psychophysiological assessment to measure the processing of unpleasant stimuli in individuals who have been exposed to maltreatment could improve the early identification of individuals at higher risk for developing anxiety symptoms. This in turn can improve the development of targeted and personalized preventive interventions for vulnerable individuals. For example, there is promising evidence that increased sensitivity to unpleasant stimuli can be modulated by psycho-social interventions to improve emotional and social well-being, address practical problems with housing and schooling, and improve coping with stressful situations in maltreated adolescents (Pincham et al., 2016). Indeed, one study showed that LPP to unpleasant stimuli was reduced following an intervention based on the prevision of support in five areas: education (e.g., avoiding school exclusion), emotion (e.g., providing a safe and confidential environment), housing (e.g., helping to find a safe and stable housing situation), legal (e.g., helping with immigration issues) and physical health (e.g., providing sexual health information) (Pincham et al., 2016). Instead, the P300/LPP complex is thought to be less sensitive to changes in response to pleasant stimuli as a function of treatment (Barch et al., 2020). On this basis, it is reasonable to assert that the P300/LPP complex may be a useful measure to integrate into the evaluation of development and therapeutic change (Pincham et al., 2016).

There are several limitations to the present results. First, the sample consisted of a population of high-functioning young adults (i.e., university students) with relatively low exposure to early childhood stress and rates of internalizing symptoms below population levels (Kessler et al., 2005). Indeed, the present study's sample reported relatively low exposure to ACEs, with 65 % endorsing at least one ACE and 37 % reporting two or more. These percentages align with the population, as suggested by a recent large meta-analysis conducted on 206 studies (Madigan et al., 2023). As such, while these findings contribute to the literature, they may not fully capture the potential associations in populations with higher exposure to early adversity. It is possible that these associations would be more pronounced in samples with higher rates of early childhood stress, and further research with more diverse samples is needed to better understand the role of adversity in emotional reactivity and internalizing symptoms. Second, it was not possible to separate the effects of maltreatment itself from numerous potential confounding factors associated with maltreatment, such as parental depression, parenting styles, genetic factors, individual differences in emotion regulation, or proximal stress exposure. In addition, due to the cross-sectional design of this study, it was not possible to establish a causal relationship between ACEs and anxiety or to understand the development and trajectory of these aberrant neural responses in the development of psychopathology in maltreated individuals. Overall, future prospective studies that include and explore larger cohorts are warranted to improve our understanding of the relationship between early adversities, emotional processing, and internalizing symptoms. Future work should aim to develop and test preventive interventions aimed at reducing the processing of unpleasant stimuli in individuals who have been exposed to ACEs. Lastly, in the current study, childhood adversities were assessed using the ACE-Q. Although this self-report has been widely employed to evaluate ACEs, it does not capture critical dimensions such as severity, chronicity, timing, or subjective impact (McLaughlin and Sheridan, 2016). These limitations may be particularly relevant given the present study's focus on understanding how early adversity interacts with neural responses to determine internalizing symptoms. The absence of dimensional information may have constrained our ability to detect associations with depressive symptoms, especially given growing evidence that depression may be more strongly influenced by the type (e.g., neglect vs. abuse) and timing of adversity (e.g., Dahl et al., 2017; Kim et al., 2021). In contrast, anxiety symptoms may emerge at lower or more uniform thresholds of threat exposure, potentially explaining why significant effects were observed for anxiety but not depression. Thus, future studies based on the dimensional model of adversity, which attempts to distil complex adverse experiences into core underlying dimensions that cut across multiple forms of adversity, may help clarify how different adversity profiles relate to specific patterns of emotional reactivity and psychopathology (McLaughlin and Sheridan, 2016). To do so, future research should rely on continuous measures of ACEs (Humphreys and Salo, 2020; McLennan et al., 2020). Taken together, the correlational design of this study, in addition to the described sample characteristics, suggests that the findings should be interpreted cautiously and replicability in non-student populations with greater ACEs is warranted.

To summarize, the present study adds to the current literature investigating how ACEs influence neural responses to affective stimuli and the role they play in determining the presence of anxiety and depressive symptoms. The results indicate that ACEs are associated with greater processing of unpleasant stimuli and less engagement and processing of pleasant stimuli. Furthermore, the present findings add to the existing literature suggesting that ACEs may be associated with the presence of anxiety symptoms in young adulthood via enhanced neural responses to unpleasant stimuli, the latter of which may act as a vulnerability factor for the development of anxiety symptoms in individuals who have been exposed to early adversities. This work has important clinical significance as the processing of unpleasant stimuli may be a target for early interventions aimed at reducing vulnerability to anxiety in maltreated individuals.

## CRediT authorship contribution statement

**Carola Dell’Acqua:** Writing – original draft, Visualization, Validation, Software, Project administration, Methodology, Investigation, Formal analysis, Data curation. **Claudio Imperatori:** Writing – review & editing, Supervision, Investigation, Funding acquisition, Formal analysis, Conceptualization. **Rita B. Ardito:** Writing – review & editing, Supervision, Investigation, Conceptualization. **Benedetto Farina:** Writing – review & editing, Investigation, Funding acquisition, Conceptualization. **Mauro Adenzato:** Writing – review & editing, Supervision, Investigation, Conceptualization. **Giuseppe Carbone:** Writing – review & editing, Investigation. **Aurelia Lo Presti:** Writing – review & editing, Investigation. **Daniela Palomba:** Writing – review & editing, Supervision, Methodology, Investigation. **Simone Messerotti Benvenuti:** Writing – review & editing, Supervision, Resources, Project administration, Investigation, Data curation, Conceptualization.

## Data availability statement

Data or other materials are available through correspondence with the first author.

## Declaration of generative IA use

During the preparation of this work, the authors used ChatGPT for language editing. After using this tool/service, the authors reviewed and edited the content as needed and take full responsibility for the content of the published article.

## Funding source

We acknowledge financial support under the National Recovery and Resilience Plan (NRRP) funded by the 10.13039/501100000780European Union – NextGenerationEU– Project Titles (“*Cognitive, affective, and neural mechanisms of depression vulnerability: searching for endophenotypes and risk factors” & “Pathways to Anhedonia: deconstruCTIng Vulnerability and training the Appetitive sysTEm in the brain”)* and number (20228P4H2K & P20223PTH4) and adopted by the Italian Ministry of University and Research (MUR).

## Declaration of competing interest

The Authors have nothing to declare.

## Footnotes

1 The IAPS picture numbers were as follows: 1050, 1114, 1120, 1300, 1302, 1930, 1932, 3500, 4611, 4647, 4651, 4652, 4660, 4664, 4670, 4680, 4683, 4690, 4695, 4810, 6200, 6210, 6230, 6242, 6243, 6244, 6250, 6260, 6312, 6313, 6370, 6510, 6540, 6550, 6560, 7000, 7002, 7004, 7009, 7010, 7020, 7035, 7036, 7041, 7050, 7056, 7059, 7130, 7175, 7224, 7233, 7242, 7491, 7500, 7547, 7560, 7595, 7700, 7950, 8030, 8031, 8034, 8080, 8161, 8180, 8185, 8186, 8200, 8370, 8400, 8490, 9425.

2 This model was conducted on differential scores (i.e., pleasant – neutral; unpleasant – neutral). However, the authors also ran the model including all three categories separately, and the results remained unchanged.

## References

[bib137] Alshogran O.Y., Altawalbeh S.M., Khalil A.A. (2022). Comparison of two self-report scales to assess anxiety and depressive symptoms in hemodialysis patients. Archiv. Psych. Nurs..

[bib1] Auerbach R.P., Bigda-Peyton J.S., Eberhart N.K., Webb C.A., Ho M.-H.R. (2011). Conceptualizing the prospective relationship between social support, stress, and depressive symptoms among adolescents. J. Abnorm. Child Psychol..

[bib2] Auerbach R.P., Stanton C.H., Proudfit G.H., Pizzagalli D.A. (2015). Self-referential processing in depressed adolescents: a high-density event-related potential study. J. Abnorm. Psychol..

[bib3] Barch D.M., Whalen D., Gilbert K., Kelly D., Kappenman E.S., Hajcak G., Luby J.L. (2020). Neural indicators of anhedonia: predictors and mechanisms of treatment change in a randomized clinical trial in early childhood depression. Biol. Psychiatry.

[bib4] Bassani L., Antypa N., Serretti A. (2013). Childhood maltreatment and neurobiological vulnerability to depression: a review. Clin. Neuropsychiatry.

[bib5] Bassani L., Antypa N., Serretti A. (2013). Childhood maltreatment and neurobiological vulnerability to depression: a review. Clin. Neuropsychiatry.

[bib139] Bates D., Mächler M., Bolker B., Walker S. (2015). Fitting linear mixed-effects models using lme4. J. Statist. Softw..

[bib6] Bechor M., Ramos M.L., Crowley M.J., Silverman W.K., Pettit J.W., Reeb-Sutherland B.C. (2019). Neural correlates of attentional processing of threat in youth with and without anxiety disorders. J. Abnorm. Child Psychol..

[bib7] Benau E.M., Hill K.E., Atchley R.A., O'Hare A.J., Gibson L.J., Hajcak G. (2019). Increased neural sensitivity to self‐relevant stimuli in major depressive disorder. Psychophysiology.

[bib8] Boecker-Schlier R., Holz N.E., Buchmann A.F., Blomeyer D., Plichta M.M., Jennen-Steinmetz C., Wolf I., Baumeister S., Treutlein J., Rietschel M. (2016). Interaction between COMT Val158Met polymorphism and childhood adversity affects reward processing in adulthood. Neuroimage.

[bib9] Bombay A., Matheson K., Anisman H. (2011). The impact of stressors on second generation Indian residential school survivors. Transcult. Psychiatry.

[bib10] Bradley M.M., Codispoti M., Cuthbert B.N., Lang P.J. (2001). Emotion and motivation I: defensive and appetitive reactions in picture processing. Emotion.

[bib11] Bradley M.M., Lang P.J. (1994). Measuring emotion: the self-assessment manikin and the semantic differential. J. Behav. Ther. Exp. Psychiatr..

[bib12] Buodo G., Sarlo M., Poli S., Giada F., Madalosso M., Rossi C., Palomba D. (2012). Emotional anticipation rather than processing is altered in patients with vasovagal syncope. Clin. Neurophysiol..

[bib13] Burani K., Klawohn J., Levinson A.R., Klein D.N., Nelson B.D., Hajcak G. (2021). Neural response to rewards, stress and sleep interact to prospectively predict depressive symptoms in adolescent girls. J. Clin. Child Adolesc. Psychol..

[bib14] Burkhouse K.L., Owens M., Feurer C., Sosoo E., Kudinova A., Gibb B.E. (2017). Increased neural and pupillary reactivity to emotional faces in adolescents with current and remitted major depressive disorder. Soc. Cognit. Affect Neurosci..

[bib15] Bylsma L.M., Morris B.H., Rottenberg J. (2008). A meta-analysis of emotional reactivity in major depressive disorder. Clin. Psychol. Rev..

[bib16] Bylsma L.M. (2021). Emotion context insensitivity in depression: toward an integrated and contextualized approach. Psychophysiology.

[bib17] Bylsma L.M., Tan P.Z., Silk J.S., Forbes E.E., McMakin D.L., Dahl R.E., Ryan N.D., Ladouceur C.D. (2022). The late positive potential during affective picture processing: associations with daily life emotional functioning among adolescents with anxiety disorders. Int. J. Psychophysiol..

[bib18] Cohen J. (2013).

[bib19] Cisler J.M., Koster E.H.W. (2010). Mechanisms of attentional biases towards threat in anxiety disorders: an integrative review. Clin. Psychol. Rev..

[bib21] Cyr C., Michel G., Dumais M. (2013). Child maltreatment as a global phenomenon: from trauma to prevention. Int. J. Psychol..

[bib22] Dahl S.K., Larsen J.T., Petersen L., Ubbesen M.B., Mortensen P.B., Munk-Olsen T., Musliner K.L. (2017). Early adversity and risk for moderate to severe unipolar depressive disorder in adolescence and adulthood: a register-based study of 978,647 individuals. J. Affect. Disord..

[bib23] De Leo D., Frisoni G.B., Rozzini R., Trabucchi M. (1993). Italian community norms for the Brief Symptom Inventory in the elderly. Br. J. Clin. Psychol..

[bib132] Del Giudice M. (2018). Evolutionary psychopathology: A unified approach.

[bib24] Del Popolo Cristaldi F., Mento G., Buodo G., Sarlo M. (2022). Emotion regulation strategies differentially modulate neural activity across affective prediction stages: an HD-EEG investigation. Front. Behav. Neurosci..

[bib25] Dell'Acqua C., Brush C.J., Burani K., Santopetro N.J., Klawohn J., Messerotti Benvenuti S., Hajcak G. (2022). Reduced electrocortical responses to pleasant pictures in depression: a brief report on time-domain and time-frequency delta analyses. Biol. Psychol..

[bib26] Dell'Acqua C., Moretta T., Dal Bò E., Messerotti Benvenuti S., Palomba D. (2022). Emotional processing prospectively modulates the impact of anxiety on COVID-19 pandemic-related post-traumatic stress symptoms: an ERP study. J. Affect. Disord..

[bib27] Delorme A., Makeig S. (2004). EEGLAB: an open source toolbox for analysis of single-trial EEG dynamics including independent component analysis. J. Neurosci. Methods.

[bib28] Dennison M.J., Rosen M.L., Sambrook K.A., Jenness J.L., Sheridan M.A., McLaughlin K.A. (2019). Differential associations of distinct forms of childhood adversity with neurobehavioral measures of reward processing: a developmental pathway to depression. Child Dev..

[bib29] Derogatis L.R., Melisaratos N. (1983). The brief symptom inventory: an introductory report. Psychol. Med..

[bib31] Dillon D.G., Holmes A.J., Birk J.L., Brooks N., Lyons-Ruth K., Pizzagalli D.A. (2009). Childhood adversity is associated with left basal ganglia dysfunction during reward anticipation in adulthood. Biol. Psychiatry.

[bib32] Ditzen B., Heinrichs M. (2014). Psychobiology of social support: the social dimension of stress buffering. Restor. Neurol. Neurosci..

[bib33] Eldar S., Yankelevitch R., Lamy D., Bar-Haim Y. (2010). Enhanced neural reactivity and selective attention to threat in anxiety. Biol. Psychol..

[bib135] Faul F., Erdfelder E., Buchner A., Lang A.G. (2009). Statistical power analyses using G∗ Power 3.1: Tests for correlation and regression analyses. Behav. Res. Meth..

[bib34] Felitti V.J., Anda R.F., Nordenberg D., Williamson D.F., Spitz A.M., Edwards V., Marks J.S. (1998). Relationship of childhood abuse and household dysfunction to many of the leading causes of death in adults: the Adverse Childhood Experiences (ACE) Study. Am. J. Prev. Med..

[bib35] Ferrara P., Corsello G., Basile M.C., Nigri L., Campanozzi A., Ehrich J., Pettoello-Mantovani M. (2015). The economic burden of child maltreatment in high income countries. J. Pediatr..

[bib36] Fitton L., Yu R., Fazel S. (2020). Childhood maltreatment and violent outcomes: a systematic review and meta-analysis of prospective studies. Trauma Violence Abuse.

[bib37] Fonzo G.A., Ramsawh H.J., Flagan T.M., Simmons A.N., Sullivan S.G., Allard C.B., Paulus M.P., Stein M.B. (2016). Early life stress and the anxious brain: evidence for a neural mechanism linking childhood emotional maltreatment to anxiety in adulthood. Psychol. Med..

[bib38] Foti D., Olvet D.M., Klein D.N., Hajcak G. (2010). Reduced electrocortical response to threatening faces in major depressive disorder. Depress. Anxiety.

[bib141] Fox, J., Weisberg, S., Price, B., Adler, D., Bates, D., Baud-Bovy, G., Bolker, B., 2019. car: Companion to Applied Regression. R package version 3.0-2. Website https://CRAN.R*-*project.org/package=car [accessed 17 March 2020]*.*

[bib39] Gardner M.J., Thomas H.J., Erskine H.E. (2019). The association between five forms of child maltreatment and depressive and anxiety disorders: a systematic review and meta-analysis. Child Abuse Negl..

[bib40] Gardner M.J., Thomas H.J., Erskine H.E. (2019). The association between five forms of child maltreatment and depressive and anxiety disorders: a systematic review and meta-analysis. Child Abuse Negl..

[bib41] Gerin M.I., Hanson E., Viding E., McCrory E.J. (2019). A review of childhood maltreatment, latent vulnerability and the brain: implications for clinical practice and prevention. Adopt. Foster..

[bib42] Gilbert K.E. (2012). The neglected role of positive emotion in adolescent psychopathology. Clin. Psychol. Rev..

[bib43] Gilbert R., Fluke J., O'Donnell M., Gonzalez-Izquierdo A., Brownell M., Gulliver P., Janson S., Sidebotham P. (2012). Child maltreatment: variation in trends and policies in six developed countries. Lancet.

[bib44] Goff B., Gee D.G., Telzer E.H., Humphreys K.L., Gabard-Durnam L., Flannery J., Tottenham N. (2013). Reduced nucleus accumbens reactivity and adolescent depression following early-life stress. Neuroscience.

[bib146] Gottesman I.I., Gould T.D. (2003). The endophenotype concept in psychiatry: etymology and strategic intentions. Am. J. Psych..

[bib47] Green J.G., McLaughlin K.A., Berglund P.A., Gruber M.J., Sampson N.A., Zaslavsky A.M., Kessler R.C. (2010). Childhood adversities and adult psychiatric disorders in the national comorbidity survey replication I: associations with first onset of DSM-IV disorders. Arch. Gen. Psychiatry.

[bib48] Guo L., Wang W., Li W., Zhao M., Wu R., Lu C. (2021). Childhood maltreatment predicts subsequent anxiety symptoms among Chinese adolescents: the role of the tendency of coping styles. Transl. Psychiatry.

[bib49] Hajcak G., Dunning J.P., Foti D. (2009). Motivated and controlled attention to emotion: time-course of the late positive potential. Clin. Neurophysiol..

[bib50] Hajcak G., Foti D. (2020). Significance?& Significance! Empirical, methodological, and theoretical connections between the late positive potential and P300 as neural responses to stimulus significance: an integrative review. Psychophysiology.

[bib51] Hanson J.L., Albert D., Iselin A.-M.R., Carre J.M., Dodge K.A., Hariri A.R. (2016). Cumulative stress in childhood is associated with blunted reward-related brain activity in adulthood. Soc. Cognit. Affect Neurosci..

[bib52] Hedrick M.J., Bonnagio T., Sellers E.W., Clements A.D. (2024). The cognitive tasks and event-related potentials associated childhood adversity: a systematic review. Neurosci. Biobehav. Rev..

[bib53] Hein T.C., Monk C.S. (2017). Research Review: neural response to threat in children, adolescents, and adults after child maltreatment–a quantitative meta‐analysis. JCPP (J. Child Psychol. Psychiatry).

[bib54] Hepp J., Schmitz S.E., Urbild J., Zauner K., Niedtfeld I. (2021). Childhood maltreatment is associated with distrust and negatively biased emotion processing. Borderline Pers. Disord. Emot. Dysregulation.

[bib55] Huang J., Wu H., Sun X., Qi S. (2023). The impact of threat of shock-induced anxiety on alerting, orienting, and executive function in women: an ERP study. Cognit. Affect Behav. Neurosci..

[bib56] Humphreys K.L., Salo V.C. (2020). Expectable environments in early life. Curr. Opin. Behav. Sci..

[bib57] Hill K.E., South S.C., Egan R.P., Foti D. (2019). Abnormal emotional reactivity in depression: contrasting theoretical models using neurophysiological data. Biol. Psychol..

[bib58] Jaffee S.R. (2017). Child maltreatment and risk for psychopathology in childhood and adulthood. Annu. Rev. Clin. Psychol..

[bib59] Jovanovic T., Norrholm S.D., Fennell J.E., Keyes M., Fiallos A.M., Myers K.M., Davis M., Duncan E.J. (2009). Posttraumatic stress disorder may be associated with impaired fear inhibition: relation to symptom severity. Psychiatry Res..

[bib60] Kamkar N.H., Lewis D.J., van den Bos W., Morton J.B. (2017). Ventral striatal activity links adversity and reward processing in children. Dev. Cogn. Neurosci..

[bib61] Kampa M., Stark R., Klucken T. (2024). The impact of past childhood adversity and recent life events on neural responses during fear conditioning. J. Neuroimaging.

[bib62] Kasparek S.W., Gastón-Panthaki A., Hanford L.C., Lengua L.J., Sheridan M.A., McLaughlin K.A. (2023). Does reward processing moderate or mediate the link between childhood adversity and psychopathology: a longitudinal study. Dev. Psychopathol..

[bib148] Kessler R.C., Berglund P., Demler O., Jin R., Merikangas K.R., Walters E.E. (2005). Lifetime prevalence and age-of-onset distributions of DSM-IV disorders in the National Comorbidity Survey Replication. Archiv. Gen. Psych..

[bib136] Khalil A.A., Hall L.A., Moser D.K., Lennie T.A., Frazier S.K. (2011). The psychometric properties of the Brief Symptom Inventory depression and anxiety subscales in patients with heart failure and with or without renal dysfunction. Archiv. Psych. Nurs..

[bib63] Kim H.-Y. (2016). Statistical notes for clinical researchers: sample size calculation 3. Comp. Several Means Using One-way ANOVA.

[bib64] Kim I., Galván A., Kim N. (2021). Independent and cumulative impacts of adverse childhood experiences on adolescent subgroups of anxiety and depression. Child. Youth Serv. Rev..

[bib65] Klawohn J., Burani K., Bruchnak A., Santopetro N., Hajcak G. (2021). Reduced neural response to reward and pleasant pictures independently relate to depression. Psychol. Med..

[bib66] Klorman R., Cicchetti D., Thatcher J.E., Ison J.R. (2003). Acoustic startle in maltreated children. J. Abnorm. Child Psychol..

[bib67] Koppold A., Kastrinogiannis A., Kuhn M., Lonsdorf T.B. (2023). Watching with Argus eyes: characterization of emotional and physiological responding in adults exposed to childhood maltreatment and/or recent adversity. Psychophysiology.

[bib68] Kok A. (2001). On the utility of P3 amplitude as a measure of processing capacity. Psychophysiology.

[bib69] Kujawa A., Burkhouse K.L. (2017). Vulnerability to depression in youth: advances from affective neuroscience. Biol. Psychiatry Cogn. Neurosci. Neuroimaging.

[bib70] Kujawa A., MacNamara A., Fitzgerald K.D., Monk C.S., Phan K.L. (2015). Enhanced neural reactivity to threatening faces in anxious youth: evidence from event-related potentials. J. Abnorm. Child Psychol..

[bib71] Krueger R.F., Markon K.E. (2006). Understanding psychopathology: melding behavior genetics, personality, and quantitative psychology to develop an empirically based model. Curr. Dir. Psychol. Sci..

[bib140] Kuznetsova A., Brockhoff P.B., Christensen R.H. (2017). lmerTest package: tests in linear mixed effects models. J. Statist. Softw..

[bib145] Lang P.J., Bradley M.M. (2010). Emotion and the motivational brain. Biol. Psychol..

[bib72] Lang P.J., Bradley M.M., Cuthbert, Greenwald M., Dhman A., Vaid D., Hamm A., Cook E., Bertron A., Petry M., Bruner R., Mcmanis M., Zabaldo D., Martinet S., Cuthbert S., Ray D., Koller K., Kolchakian M., Hayden S. (1997). International Affective Picture System.

[bib73] LeMoult J., Gotlib I.H. (2019). Depression: a cognitive perspective. Clin. Psychol. Rev..

[bib147] Lenzenweger M.F. (2013). Thinking clearly about the endophenotype–intermediate phenotype–biomarker distinctions in developmental psychopathology research. Dev. Psychopathol..

[bib74] Luck S.J. (2014).

[bib75] Luckhardt C., Mühlherr A.M., Schütz M., Jarczok T.A., Jungmann S.M., Howland V., Veit L., Althen H., Freitag C.M. (2023). Reward processing in adolescents with social phobia and depression. Clin. Neurophysiol..

[bib76] Luking K.R., Pagliaccio D., Luby J.L., Barch D.M. (2016). Reward processing and risk for depression across development. Trends Cognit. Sci..

[bib133] Luyten P., Fonagy P. (2018). The stress–reward–mentalizing model of depression: An integrative developmental cascade approach to child and adolescent depressive disorder based on the Research Domain Criteria (RDoC) approach. Clin. Psychol. Rev..

[bib77] MacNamara A., Hajcak G. (2010). Distinct electrocortical and behavioral evidence for increased attention to threat in generalized anxiety disorder. Depress. Anxiety.

[bib78] Madigan S., Deneault A.A., Racine N., Park J., Thiemann R., Zhu J. (2023). Adverse childhood experiences: a meta‐analysis of prevalence and moderators among half a million adults in 206 studies. World Psychiatry.

[bib79] MacNamara A., Kotov R., Hajcak G. (2016). Diagnostic and symptom-based predictors of emotional processing in generalized anxiety disorder and major depressive disorder: an event-related potential study. Cognit. Ther. Res..

[bib80] Massullo C., De Rossi E., Carbone G.A., Imperatori C., Ardito R.B., Adenzato M., Farina B. (2023). Child maltreatment, abuse, and neglect: an umbrella review of their prevalence and definitions. Clin. neuropsychiatry.

[bib82] Matz K., Junghöfer M., Elbert T., Weber K., Wienbruch C., Rockstroh B. (2010). Adverse experiences in childhood influence brain responses to emotional stimuli in adult psychiatric patients. Int. J. Psychophysiol..

[bib83] McLaughlin K.A., DeCross S.N., Jovanovic T., Tottenham N. (2019). Mechanisms linking childhood adversity with psychopathology: learning as an intervention target. Behav. Res. Ther..

[bib84] McLaughlin K.A., Peverill M., Gold A.L., Alves S., Sheridan M.A. (2015). Child maltreatment and neural systems underlying emotion regulation. J. Am. Acad. Child Adolesc. Psychiatr..

[bib85] McLaughlin K.A., Sheridan M.A. (2016). Beyond cumulative risk: a dimensional approach to childhood adversity. Curr. Dir. Psychol. Sci..

[bib86] McLennan J.D., MacMillan H.L., Afifi T.O. (2020). Questioning the use of adverse childhood experiences (ACEs) questionnaires. Child Abuse Negl..

[bib143] Messerotti Benvenuti S., Buodo G., Dal Bò E., Palomba D. (2020). Attention and affect in dysphoria: Insights from startle reflex modulation and cardiac deceleration. Behav. Res. Therap..

[bib87] Michelini G., Perlman G., Tian Y., Mackin D.M., Nelson B.D., Klein D.N., Kotov R. (2021). Multiple domains of risk factors for first onset of depression in adolescent girls. J. Affect. Disord..

[bib88] Moog N.K., Cummings P.D., Jackson K.L., Aschner J.L., Barrett E.S., Bastain T.M., Blackwell C.K., Enlow M.B., Breton C.V., Bush N.R. (2023). Intergenerational transmission of the effects of maternal exposure to childhood maltreatment in the USA: a retrospective cohort study. Lancet Public Health.

[bib89] Moretta T., Dal Bò E., Dell'Acqua C., Messerotti Benvenuti S., Palomba D. (2021). Disentangling emotional processing in dysphoria: an ERP and cardiac deceleration study. Behav. Res. Ther..

[bib90] Moretta T., Messerotti Benvenuti S. (2023). Familial risk for depression is associated with reduced P300 and late positive potential to affective stimuli and prolonged cardiac deceleration to unpleasant stimuli. Sci. Rep..

[bib91] Nelson B.D., Perlman G., Klein D.N., Kotov R., Hajcak G. (2016). Blunted neural response to rewards as a prospective predictor of the development of depression in adolescent girls. Am. J. Psychiatr..

[bib92] Norman R.E., Byambaa M., De R., Butchart A., Scott J., Vos T. (2012). The long-term health consequences of child physical abuse, emotional abuse, and neglect: a systematic review and meta-analysis. PLoS Med..

[bib93] Novak B.K., Novak K.D., Lynam D.R., Foti D. (2016). Individual differences in the time course of reward processing: stage-specific links with depression and impulsivity. Biol. Psychol..

[bib94] Novak K.D., Foti D. (2015). Teasing apart the anticipatory and consummatory processing of monetary incentives: an event-related potential study of reward dynamics. Psychophysiology.

[bib95] Olino T.M., McMakin D.L., Morgan J.K., Silk J.S., Birmaher B., Axelson D.A., Williamson D.E., Dahl R.E., Ryan N.D., Forbes E.E. (2014). Reduced reward anticipation in youth at high-risk for unipolar depression: a preliminary study. Dev. Cogn. Neurosci..

[bib96] Oltean L.-E., Șoflău R., Miu A.C., Szentágotai-Tătar A. (2023). Childhood adversity and impaired reward processing: a meta-analysis. Child Abuse Negl..

[bib97] Palomba D., Angrilli A., Mini A. (1997). Visual evoked potentials, heart rate responses and memory to emotional pictorial stimuli. Int. J. Psychophysiol..

[bib98] Park I., Oh S.M., Lee K.H., Kim S., Jeon J.E., Lee H.Y., Jeon S., Kim S.J., Lee Y.J. (2020). The moderating effect of sleep disturbance on the association of stress with impulsivity and depressed mood. Psychiatr. Invest..

[bib99] Pedersen W.S., Larson C.L. (2016). State anxiety carried over from prior threat increases late positive potential amplitude during an instructed emotion regulation task. Emotion.

[bib100] Pincham H.L., Bryce D., Kokorikou D., Fonagy P., Fearon R.M.P. (2016). Psychosocial intervention is associated with altered emotion processing: an event-related potential study in at-risk adolescents. PLoS One.

[bib101] Pizzagalli D.A. (2014). Depression, stress, and anhedonia: toward a synthesis and integrated model. Annu. Rev. Clin. Psychol..

[bib102] Poli S., Sarlo M., Bortoletto M., Buodo G., Palomba D. (2007). Stimulus-preceding negativity and heart rate changes in anticipation of affective pictures. Int. J. Psychophysiol..

[bib103] Polich J. (2007). Updating P300: an integrative theory of P3a and P3b. Clin. Neurophysiol..

[bib104] Pollak S.D., Cicchetti D., Klorman R., Brumaghim J.T. (1997). Cognitive brain event-related potentials and emotion processing in maltreated children. Child Dev..

[bib105] Pollak S.D., Sinha P. (2002). Effects of early experience on children's recognition of facial displays of emotion. Dev. Psychol..

[bib106] Pollak S.D., Tolley-Schell S.A. (2003). Selective attention to facial emotion in physically abused children. J. Abnorm. Psychol..

[bib107] Poole J.C., Dobson K.S., Pusch D. (2018). Do adverse childhood experiences predict adult interpersonal difficulties? The role of emotion dysregulation. Child Abuse Negl..

[bib108] Quevedo K., Johnson A.E., Loman M.M., Lafavor T., Moua B., Gunnar M.R. (2015). The impact of early neglect on defensive and appetitive physiology during the pubertal transition: a study of startle and postauricular reflexes. Dev. Psychobiol..

[bib138] Riskind J.H., Kleiman E.M., Seifritz E., Neuhoff J. (2014). Influence of anxiety, depression and looming cognitive style on auditory looming perception. J. Anxiety Disorders.

[bib109] Rottenberg J., Gross J.J., Gotlib I.H. (2005). Emotion context insensitivity in major depressive disorder. J. Abnorm. Psychol..

[bib110] Saarinen A., Keltikangas-Järvinen L., Jääskeläinen E., Huhtaniska S., Pudas J., Tovar-Perdomo S., Penttilä M., Miettunen J., Lieslehto J. (2021). Early adversity and emotion processing from faces: a meta-analysis on behavioral and neurophysiological responses. Biol. Psychiatry Cogn. Neurosci. Neuroimaging.

[bib111] Sandre A., Ethridge P., Kim I., Weinberg A. (2018). Childhood maltreatment is associated with increased neural response to ambiguous threatening facial expressions in adulthood: evidence from the late positive potential. Cognit. Affect Behav. Neurosci..

[bib112] Schupp H.T., Cuthbert B.N., Bradley M.M., Cacioppo J.T., Ito T., Lang P.J. (2000). Affective picture processing: the late positive potential is modulated by motivational relevance. Psychophysiology.

[bib113] Sethi D., Bellis M., Hughes K., Gilbert R., Mitis F., Galea G. (2013).

[bib114] Shackman J.E., Shackman A.J., Pollak S.D. (2007). Physical abuse amplifies attention to threat and increases anxiety in children. Emotion.

[bib115] Sherdell L., Waugh C.E., Gotlib I.H. (2012). Anticipatory pleasure predicts motivation for reward in major depression. J. Abnorm. Psychol..

[bib116] Simons R.F., Öhman A., Lang P.J. (1979). Anticipation and response set: cortical, cardiac, and electrodermal correlates. Psychophysiology.

[bib117] Speed B.C., Nelson B.D., Auerbach R.P., Klein D.N., Hajcak G. (2016). Depression risk and electrocortical reactivity during self-referential emotional processing in 8 to 14 year-old girls. J. Abnorm. Psychol..

[bib118] Sussman T.J., Szekely A., Hajcak G., Mohanty A. (2016). It's all in the anticipation: how perception of threat is enhanced in anxiety. Emotion.

[bib119] Stoltenborgh M., Bakermans‐Kranenburg M.J., Alink L.R.A., van Ijzendoorn M.H. (2015). The prevalence of child maltreatment across the globe: review of a series of meta‐analyses. Child Abuse Rev..

[bib120] Tadel F., Baillet S., Mosher J.C., Pantazis D., Leahy R.M. (2011). Brainstorm: a user-friendly application for MEG/EEG analysis. Comput. Intell. Neurosci..

[bib121] Takeuchi S., Mochizuki Y., Masaki H., Takasawa N., Yamazaki K. (2005). Stimulus preceding negativity represents arousal induced by affective picture. Int. Congr. Ser..

[bib122] Takiguchi S., Fujisawa T.X., Mizushima S., Saito D.N., Okamoto Y., Shimada K., Koizumi M., Kumazaki H., Jung M., Kosaka H. (2015). Ventral striatum dysfunction in children and adolescents with reactive attachment disorder: functional MRI study. BJPsych. Open.

[bib123] Taylor S.E. (2011). Social support: a review. Oxf. Handb. Health Psychol..

[bib142] Thompson B., Santopetro N.J., Brush C.J., Foti D., Hajcak G. (2023). Neural deficits in anticipatory and consummatory reward processing are uniquely associated with current depressive symptoms during adolescence. Psychophysiology.

[bib124] Turner R.J., Brown R.L. (2010). Social support and mental health. Handb. Stud. Mental Health: Social Contexts, Theori. Sys..

[bib125] Umemoto A., Holroyd C.B. (2017). Neural mechanisms of reward processing associated with depression-related personality traits. Clin. Neurophysiol..

[bib126] Van Bockstaele B., Verschuere B., Tibboel H., De Houwer J., Crombez G., Koster E.H.W. (2014). A review of current evidence for the causal impact of attentional bias on fear and anxiety. Psychol. Bull..

[bib134] Wang D., Liu T., Shi J. (2020). Neural dynamic responses of monetary and social reward processes in adolescents. Front. Human Neurosci..

[bib127] Webb C.A., Auerbach R.P., Bondy E., Stanton C.H., Appleman L., Pizzagalli D.A. (2021). Reward-related neural predictors and mechanisms of symptom change in cognitive behavioral therapy for depressed adolescent girls. Biol. Psychiatry Cogn. Neurosci. Neuroimaging.

[bib128] Weinberg A., Venables N.C., Proudfit G.H., Patrick C.J. (2015). Heritability of the neural response to emotional pictures: evidence from ERPs in an adult twin sample. Soc. Cognit. Affect Neurosci..

[bib144] Weinberg A., Hajcak G. (2010). Beyond good and evil: the time-course of neural activity elicited by specific picture content. Emotion.

[bib129] Weinberg A., Perlman G., Kotov R., Hajcak G. (2016). Depression and reduced neural response to emotional images: distinction from anxiety, and importance of symptom dimensions and age of onset. J. Abnorm. Psychol..

[bib130] Weinberg A. (2023). Pathways to depression: dynamic associations between neural responses to appetitive cues in the environment, stress, and the development of illness. Psychophysiology.

[bib131] Zuckerman M., Zuckerman M. (1999). Vulnerability to Psychopathology: A Biosocial Model.

