# Supplementary material for: Adverse childhood experiences and internalizing symptoms: the moderating role of neural responses to threat
Source: Neurobiol Stress. 2025 Jun 10;37:100740. doi: 10.1016/j.ynstr.2025.100740 (PMC12206150; doi:10.1016/j.ynstr.2025.100740)
Supplement: Multimedia component 1 [file mmc1.docx]

**Supplementary material**

*Adverse childhood experiences and internalizing symptoms: the moderating role of neural responses to threat*

*Dell’Acqua et al.*


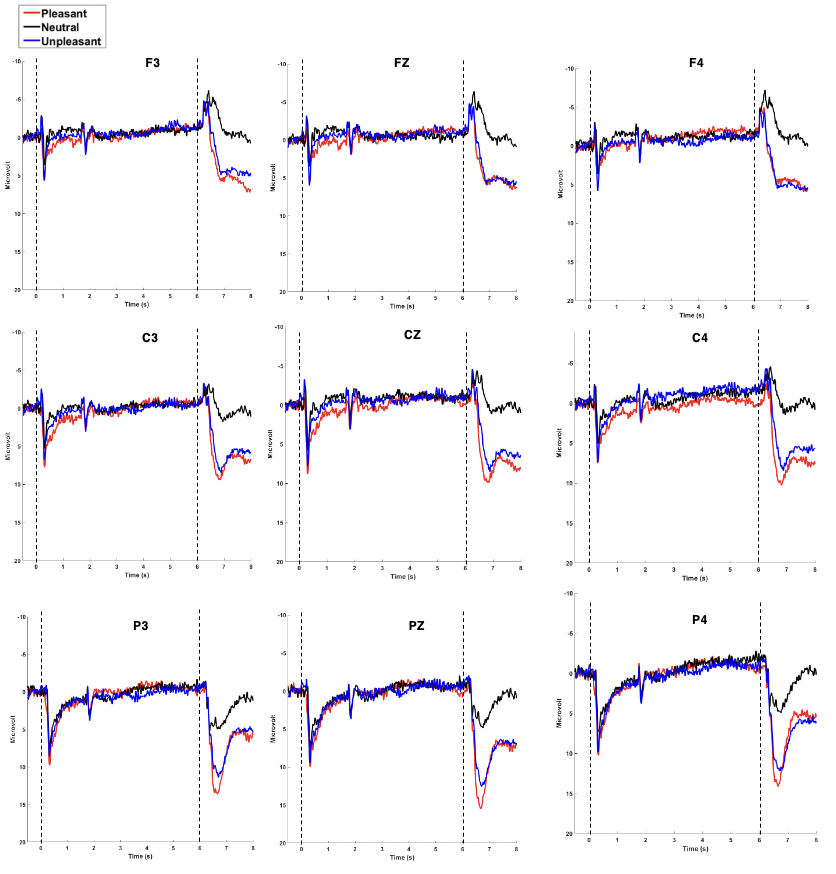


**Figure S1.** Grand average ERP waveforms during the S1-S2 task at frontal (F3, FZ, F4), central (C3, CZ, C4), and parietal (P3, PZ, P4) electrodes.
